# Circulating Biomarkers of Colorectal Cancer (CRC)—Their Utility in Diagnosis and Prognosis

**DOI:** 10.3390/jcm10112391

**Published:** 2021-05-28

**Authors:** Marta Łukaszewicz-Zając, Barbara Mroczko

**Affiliations:** 1 Department of Biochemical Diagnostics, Medical University of Bialystok, ul. Waszyngtona 15 a, 15-269 Bialystok, Poland; mroczko@umb.edu.pl; 2 Department of Neurodegeneration Diagnostics, Medical University of Bialystok, 15-269 Bialystok, Poland

**Keywords:** biomarker, colorectal cancer, tumor

## Abstract

The global burden of colorectal cancer (CRC) is expected to increase, with 2.2 million new cases and 1.1 million annual deaths by 2030. Therefore, the establishment of novel biomarkers useful in the early diagnosis of CRC is of utmost importance. A number of publications have documented the significance of the overexpression of several specific proteins, such as inflammatory mediators, in CRC progression. However, little is known about the potential utility of these proteins as circulating blood tumor biomarkers of CRC. Therefore, in the present review we report the results of our previous original studies as well as the findings of other authors who investigated whether inflammatory mediators might be used as novel biomarkers in the diagnosis and prognosis of CRC. Our study revealed that among all of the tested proteins, serum M-CSF, CXCL-8, IL-6 and TIMP-1 have the greatest value in the diagnosis and progression of CRC. Serum TIMP-1 is useful in differentiating between CRC and colorectal adenomas, whereas M-CSF and CRP are independent prognostic factors for the survival of patients with CRC. This review confirms the promising significance of these proteins as circulating biomarkers for CRC. However, due to their non-specific nature, further validation of their sensitivity and specificity is required.

## 1. Colorectal Cancer

Colorectal cancer (CRC) is one of the most common neoplasms worldwide. It is the second most frequently diagnosed malignancy in women and third in men. It is estimated that more than one million people worldwide develop CRC every year. In addition, this carcinoma is the second leading cause of cancer death in Europe, ranking fourth in males and third in females [1,2,3].

Depending on the origin of the mutation, colorectal carcinomas can be classified as sporadic, inherited, or familial [4,5]. Both genetic and environmental factors play an important role in the etiology of CRC. Over 70% of CRC cases are sporadic, hence related to lifestyle, while approximately three quarters of CRC cases are caused by the interplay of environmental and genetic factors. The incidence of CRC increases with age. The majority of patients with sporadic CRC are over 50 years old, whereas 75% of the patients with rectal cancer, and 80% of the subjects with colon cancer are over the age of 60 at the time of diagnosis [3]. Other known risk factors for this disease include a diet low in vegetables and fruit, excessive intake of red meat, saturated fats and alcohol, tobacco use, and being overweight [6,7]. A small percentage of CRC cases are due to inherited forms [6]. Gene mutations occurring in oncogenes, tumor suppressor genes and genes related to DNA repair mechanisms may lead to CRC development [4,5]. In addition, it is estimated that around 20% of CRC cases have an associated hereditary component—familial CRC [6,7,8,9]. Approximately 60% of CRC cases are diagnosed in developed countries, with Europe being a region featuring the highest incidence and mortality rates [6].

Due to the high mortality and morbidity of CRC, diagnostic methods useful in the detection of early-stage disease are sorely needed. The most significant prognostic factor for CRC is the disease stage at the time of diagnosis. Thus, the 5-year relative survival rate for patients with CRC is around 50–60%. It is higher, and stands at approximately 80%, if the tumor is detected at an early stage, and decreases below 15% for advanced-stage cancer [6,10,11]. CRC is commonly diagnosed at an advanced stage based on signs and symptoms such as blood in the stool, a change in bowel habits, abdominal pain, fatigue, anemia-related symptoms, or unintended weight loss. The highest incidence rates for CRC are observed among individuals aged 50 years and over, who are asymptomatic and do not have a personal or family history of CRC. In symptomatic patients, colonoscopy is the preferred diagnostic modality, although other endoscopic methods, such as high-definition white-light endoscopy, chromoendoscopy, autofluorescence endoscopy or narrow-band imaging, are also very useful [3]. The CRC diagnostic process is extensive, and involves the use of imaging modalities and laboratory methods. Serum carcinoembryonic antigen (CEA), which is the most extensively investigated classical tumor marker, is not useful in screening because of its low diagnostic sensitivity in early stage CRC. Other serum biomarkers, including cancer antigen 19-9 (CA 19.9), cancer antigen 50 (CA 50) or cancer antigen 72.4 (CA 72.4), have been studied without demonstrating acceptable diagnostic performance [12]. Although a number of candidates for new biochemical markers for CRC have been evaluated during the last decade, there are still no biomarkers useful in the early diagnosis of CRC. Thus, novel noninvasive diagnostic tools for the early detection of CRC, such as circulating blood markers, are sorely needed to improve the management and outcomes of patients with CRC.

**Figure 1:**
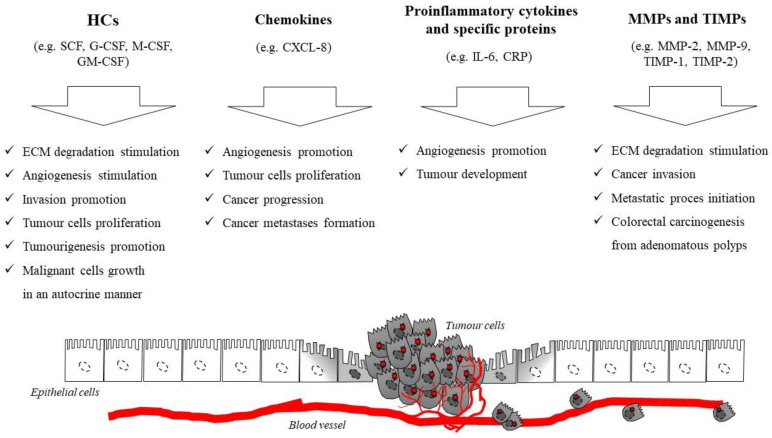
The role of selected cytokines, proinflammatory proteins, matrix metalloproteinases and their tissue inhibitors in the pathogenesis of colorectal cancer (CRC).

Therefore, in this review we present a number of research papers that explore the clinical significance of selected inflammatory mediators as potential biomarkers of CRC in comparison to well established, classical tumor markers for this malignancy. Furthermore, the review presents the results of the authors’ previous studies, which have demonstrated the diagnostic and prognostic utility of selected proteins as candidates for biochemical markers for CRC. The clinical application of biomarkers in CRC is required not only for the early detection of the disease, but also for prognostic stratification and surveillance of patients with this neoplasm. CRC develops due to the accumulation of genetic and epigenetic alterations, which leads to the transformation of normal colonic mucosa to invasive cancer [6]. The transition from normal epithelial cells to adenocarcinoma starts from a polyp with an aberrant crypt, which transforms to an early adenoma, and subsequently becomes colorectal cancer. This sequence lasts for more than a decade, although it may progress more rapidly in individuals with certain disorders such as Lynch syndrome [3,13]. Thus, the majority of colorectal carcinomas develop from adenomas (adenoma–carcinoma sequence). The Fearon–Vogelstein model pioneered the combined consideration of single, but accumulating, molecular events and biological consequences during tumor progression. There are two discrete normal colon to colorectal cancer sequences. However, both of them concern the progression of normal colon epithelial cells to aberrant crypt foci, followed by early and advanced polyps with the subsequent progression to early cancer, and then advanced CRC. The classic pathway involves the development of tubular adenomas that progress to adenocarcinomas, while an alternate pathway involves serrated polyps. Furthermore, mutated or epigenetically altered genes are indicated in both of the sequences. Some genes are shared between the two pathways while others are unique, such as BRAF mutations that occur only in the serrated pathway [3,6,13]. The period of neoplastic transformation is thought to be approximately 10–15 years, which represents the time available for the detection and removal of adenomas before they progress to invasive carcinoma [6]. It has been proven that prolonged chronic inflammation plays a key role in the pathogenesis of many malignancies, including CRC, and might stimulate the synthesis of proinflammatory mediators within the tumor microenvironment. This process contributes to tumor initiation, promotion and progression, mostly via the migration of tumor cells. Moreover, a developing neoplasm is able to induce local and systemic inflammatory responses [14,15,16,17]. Furthermore, the infiltration of various immune cells, such as tumor-infiltrating leukocytes (TILs), various cytokines and tissue remodeling factors, has also been associated with cancer-related inflammation. Our previous original studies as well as some clinical investigations of other authors prove that selected circulating inflammatory mediators such as cytokines, including chemokines and hematopoietic growth factors (HGFs), specific inflammatory proteins such as C-reactive protein (CRP) and various matrix metalloproteinases, in particular gelatinases (MMP-2, MMP-9) and their tissue inhibitors (TIMP-1, TIMP-2), could be involved in the tumor development, proliferation, migration and angiogenesis of cancer cells (Figure 1), and may be considered potential candidates for novel biochemical markers for CRC (Table 1) [18,19,20,21,22,23,24,25,26,27,28].

## 2. Cytokines

### 2.1. Hematopoietic Cytokines (HCs)

Hematopoietic cytokines (HCs), also known as hematopoietic growth factors (HGFs), are small proteins that induce the differentiation and proliferation of hematopoietic progenitor cells. Some studies have found that the effects of these growth factors are not limited to bone marrow cells—they are also able to stimulate the proliferation of non-hematopoietic and malignant cells, including CRC [18,19,20,21,29,30]. Moreover, these small peptides promote extracellular matrix (ECM) degradation and angiogenesis, thus facilitating the invasion and proliferation of tumor cells [31,32,33]. Some HCs, such as granulocyte colony-stimulating factor (G-CSF) and macrophage colony-stimulating factor (M-CSF) promote the tumorigenesis and growth of malignant cells in an autocrine manner. Furthermore, several cell lines of malignant tumors, including CRC, have been found to secrete large amounts of HCs as well as express their receptors [34,35]. A number of investigators have assessed the expression of these molecules in CRC tissue using an immunohistochemical technique in order to evaluate their role in the pathogenesis of CRC. Therefore, in our previous research we focused on evaluating the serum concentrations of several circulating HCs, including stem cell factor (SCF), granulocyte–macrophage colony-stimulating factor (GM-CSF), macrophage colony-stimulating factor (M-CSF), and interleuln-3 (IL-3), in patients with CRC in comparison to subjects with benign lesions of the colon (colorectal adenomas—CA), and healthy volunteers, in order to establish their diagnostic and prognostic value as well as to investigate a potential role played by these proteins in the pathogenesis of CRC [18,19,20,21]. In our studies, the concentrations of GM-CSF, M-CSF, and IL-3 were significantly higher, and the concentration of SCF was significantly lower in the serum of the patients with CRC in comparison to the control groups and subjects with CA [18,19]. Our results have been confirmed by other authors who also demonstrated elevated concentrations of several circulating HCs in patients with CRC in comparison to healthy individuals [35,36,37]. Furthermore, in our studies, the serum levels of classical tumor markers for this malignancy—CEA and CA 19-9—were also statistically significantly elevated compared to the control groups [18,21]. Moreover, there was a significant association between the M-CSF levels and TNM stage, as well as between the serum M-CSF levels and lymph node metastasis in the Kruskal–Wallis test [18], which suggests the role of this cytokine in the pathogenesis and progression of CRC. Among all the HCs investigated in our studies, the diagnostic sensitivity of SCF was higher than that of other cytokines and classical tumor markers. The highest percentage of positive results was obtained for the combined analysis of SCF, and GM-CSF or M-CSF (96%), or for the combined measurement of SCF and classical tumor markers (both 93%) [19]. However, the diagnostic specificity and predictive values were the highest for M-CSF among all the analyzed cytokines [18,21]. In addition, to demonstrate the diagnostic value of the investigated proteins, we compared the areas under the ROC (receiver operating characteristic) curve, AUC, for all of the measurements. The AUC for M-CSF was larger than the AUCs for SCF, IL-3, GM-CSF and CA 19-9, but similar to the AUC for CEA [18,19]. In addition, in a multivariate analysis, we showed that serum M-CSF is an independent prognostic factor for the survival of patients with CRC (*p* = 0.011) [18].

In conclusion, we compared the diagnostic value of selected HCs in patients with CRC with classical tumor markers for this malignancy—CEA and CA 19-9. In line with the results obtained by other authors, our findings revealed the diagnostic utility of selected HCs as potential, biochemical tumor markers for CRC [37,38]. Among all of the examined HCs, the highest utility was demonstrated for M-CSF and SCF in the diagnosis of CRC, particularly in combination with CEA. Moreover, our studies proved the clinical significance of serum M-CSF measurements in estimating prognosis for patients with CRC.

### 2.2. Chemokines and Their Specific Receptors

Chemokines are a family of low-molecular-weight cytokines grouped into four classes—CC, CXC, CX3C and XC—based on the positions of key cysteine residues [39]. These proteins act via the cognate G-protein-coupled seven transmembrane receptors (GPCRs) to cause adhesion, chemotaxis and migration of cells [40,41]. It has been demonstrated that these molecules play a crucial role in the regulation of leukocyte function including growth, activation and differentiation. Thus, they are able to regulate physiological processes, e.g., inflammatory, infection, immunity processes as well as pathological processes, including malignant diseases such as CRC [42]. Selected chemokines and their specific receptors facilitate tumor dissemination at every stage of carcinogenesis, i.e., progression, proliferation, adherence of cancer cells to the endothelium, extravasation from blood vessels, and angiogenesis [43,44,45]. Furthermore, these cytokines stimulate communication between malignant and non-malignant cells within the TME, and contribute to the activation of neutrophils and tumor-associated macrophages. Some investigators indicate the utility of various chemokines and their specific receptors, particularly those belonging to the CXC family, in cancer pathogenesis, including CRC [46,47,48,49,50,51,52,53,54,55,56,57,58,59,60,61,62,63,64,65].

It has been demonstrated that CXCL12 (CXC-chemokine ligand 12) and its receptors, CXCR4 and CXCR7, are involved in cancer metastasis [46,47]. It has been shown that CRC cells are able to express these proteins [48,49]. The expression of CXCL12, CXCR4 and CXCR7 is elevated in CRC and tissue samples from lung metastasis, while the expression of both CXCL12 and CXCR7 is significantly higher in tissue samples derived from metastatic cancer in comparison to primary lesions [47,50]. Some clinical investigations have demonstrated the role of other chemokines, such as CCL20 (CC-chemokine ligand 20) and its specific receptor CCR6 (CC-chemokine receptor 6), receptor CXCR3 (CXC chemokine receptor 3) as well as CXCL5 (CXC motif chemokine 5), in CRC pathogenesis [51,52,53,54,55]. The expression of CCL20 and CCR6 is elevated in CRC samples compared to non-malignant tissue [51]. The overexpression of CCR6 in CRC cells correlates with the presence of distant metastases [52], while the overexpression of CXCR3 promotes metastases to lymph nodes [53]. Moreover, the expression of CXCL10 is considered an independent prognostic factor for cancer recurrence in CRC [54], while the co-expression of both CXCR3 and CXCL10 in CRC is linked to poorer prognosis and metastatic recurrence [55].

One of the most extensively investigated chemokines is CXCL-8. This protein is involved in tumor angiogenesis and has been linked to promoting distant metastases in CRC [56]. Expression of this protein has been demonstrated on endothelial cells, tumor-associated macrophages and cancer cells, including CRC [57]. Furthermore, CXCL-8 expression is significantly higher in all CRC tissues in comparison to inflammatory and non-malignant samples [58], and correlates with the presence of distant metastases, which indicates the significance of this cytokine as a marker of CRC progression [59]. All of the results presented above have been obtained using a time-consuming immunohistochemical technique [51,52,53,54,55,56,57,58,59,60,61]. Therefore, in our most recent studies we investigated whether serum CXCL-8 and its specific receptor (CXCR-2) may be used as potential biochemical tumor markers for CRC using the enzyme-linked immunosorbent assays (ELISA) method. We compared the diagnostic utility of serum levels of CXCL-8 and CXCR-2 with classical tumor markers (CEA) for CRC [22,23,24]. Our investigations revealed that serum concentrations of CXCL-8, similar to those of the classical tumor marker, were significantly higher in patients with CRC in comparison to healthy controls—the results are consistent with those obtained by other authors [62]. These findings suggest that CRC cells are able to produce CXCL-8. We also demonstrated statistically significant differences between CXCL-8 concentrations and tumor stages, and the presence of distant metastasis (M-factor) [22]. Our observations are in line with the results obtained by other researchers who also proved that serum CXCL-8 levels significantly correlate with CRC stage [58,63]. We compared the diagnostic characteristics of CXCL-8 with those of the classical tumor markers for CRC. Diagnostic sensitivity, the predictive value of negative (NPV) results, and accuracy were higher for serum CXCL-8 when compared to CEA. Moreover, the AUC for CXCL-8 (0.778; *p* < 0.001) was higher than for CEA in patients with CRC. Based on our results, we can conclude that serum CXCL-8 is a better candidate for a biochemical marker in the diagnosis of CRC than CEA, which is the marker currently used in routine clinical practice. As a continuation of our previous research, we investigated the clinical utility of the receptor specific for CXCL-8–CXCR-2 in CRC [22,23]. The serum levels of CXCR-2 were found to be lower, while those of CEA were significantly higher in patients with CRC in comparison to healthy controls. Moreover, the diagnostic sensitivity was higher for CXCR-2 than for CEA, and increased in the combined analysis of CXCR-2 and CEA. Our results suggest that the CXCL-8/CXCR-2 axis plays an important role in the pathogenesis of CRC [22].

Some clinical investigations have indicated that CXCL5 (CXC motif chemokine 5) and CXCL15 (CXC motif chemokine 15) may also be significant in CRC progression. The serum concentrations of these chemokines were found to be significantly higher in patients with CRC in comparison to healthy volunteers [64,65]. However, the authors failed to evaluate statistically significant results between the analyzed subgroups, and concluded that serum CXCL5 cannot be recognized as a potential marker in CRC, although CXCL15 concentrations increased with disease stage and correlated with poor survival [64,65].

In summary, our previous findings as well as data reported by other investigators suggest that selected chemokines and their specific receptors are involved in CRC pathogenesis. Alterations in the levels of these proteins correlate with advanced stage, metastatic recurrence and poor survival of patients with CRC. In conclusion, CXCL-8 concentrations have been suggested as potential biochemical tumor markers, particularly in the combined assessment with a well-established classical tumor marker for CRC–CEA.

## 3. Interleukin-6 and C-Reactive Protein

Another cytokine considered a potential tumor maker for CRC is interleukin-6 (IL-6). This protein is a pleiotropic proinflammatory cytokine that plays a dual role in tumor development [66]. IL-6 promotes the apoptosis of neoplastic cells by stimulating the antitumor activity of macrophages. However, this cytokine is produced by cancer cell lines, including CRC, and may stimulate neoangiogenesis [67]. It has been established that proinflammatory cytokines such as IL-6 promote the synthesis of acute-phase proteins including C-reactive protein (CRP) [68]. Some authors suggest that malignant cells also stimulate CRP synthesis in hepatocytes [69]. Thus, a number of studies indicate that IL-6 is a mediator that links inflammation and angiogenesis to malignancy [43]. In our previous study, we examined the correlations between pretreatment serum levels of IL-6 and CRP, and the clinicopathological features of CRC, such as tumor size, the presence of distant and lymph node metastases, tumor resectability as well as the prognostic significance of these mediators in patients with CRC [25]. We also determined the serum levels of IL-6 and CRP in patients with CA, and assessed the diagnostic utility of these parameters in differentiating between CRC and CA [25]. We demonstrated that the serum concentrations of CRP and IL-6 were significantly higher in patients with CRC in comparison to patients with CA, and healthy subjects, and increased in more advanced stages of the disease and in the subjects with unresectable tumors. There was a significant difference between the serum levels of IL-6 and the presence of lymph node and distant metastases, while the CRP concentrations were statistically higher in patients with distant metastases compared to the subjects in subgroup M0. Similar results have been demonstrated by other authors who also revealed that serum IL-6 and CRP concentrations are significantly higher in patients with CRC than in healthy subjects, and increase in more advanced stages of the disease [63,70,71]. To assess the diagnostic significance of the tested proteins, we evaluated the AUC, which was found to be the highest for the IL-6 levels among all of the proteins tested (CPR, CEA and CA 19-9). Furthermore, the elevated preoperative serum level of CRP was found to be an independent significant prognostic factor for patient survival [25]. Other authors have also confirmed the significance of IL-6 in CRC development and, similarly to our findings, demonstrated that elevated preoperative CRP concentrations may serve as a predictor of the unfavorable prognosis of patients with CRC, which reflects the synthesis of acute-phase proteins during tumor progression [72]. Based on our results and data published by other investigators, we suggest the usefulness of serum IL-6 measurements in CRC diagnosis, and the utility of CRP levels in the prediction of patient survival [25].

## 4. Matrix Metalloproteinases (MMPs) and Their Tissue Inhibitors (TIMPs)

Adenomatous polyps may develop into invasive adenocarcinoma of the colon or rectum via increasing grades of dysplasia to the carcinoma in situ. Cytokines, whose role in the inflammatory process and, indirectly, in the pathogenesis of malignancies is well established, also promote ECM degradation. Some clinical investigations have demonstrated that ECM degradation plays a crucial role in the development of malignant neoplasms by controlling cancer proliferation [73]. Furthermore, cancer cells, including CRC, are able to produce and release proteolytic enzymes, such as matrix metalloproteinases (MMPs), which are capable of degrading the basement membrane and type IV collagen in the ECM [74,75]. MMPs are produced by various cells including macrophages, fibroblasts, leukocytes, endothelial cells and tumor cells. The proteolytic activity of MMPs is regulated by naturally occurring tissue inhibitors of matrix metalloproteinases (TIMPs). It has been indicated that the remodeling of normal and tumor tissue may result from an imbalance between MMPs and TIMPs [76,77,78,79] in CRC tissue, and consequently might be a significant factor in the process of cancer invasion and metastasis. Thus, MMPs and TIMPs play a role not only in CRC invasion and the initiation of the metastatic process, but also in colorectal carcinogenesis from adenomatous polyps. CRC is characterized by its enhanced expression of several MMPs such as MMP-1, MMP-2, MMP-7 or MMP-13. However, a group of MMPs—gelatinases—is of particular interest to researchers with respect to the development and progression of CRC [80]. Gelatinases, including metalloproteinase 9 (MMP-9) and metalloproteinase 2 (MMP-2), play an important role in the development of malignancies through facilitating tumor invasion, metastasis, growth, cell migration and angiogenesis. Some authors have demonstrated that the expression of MMP-2 is significantly higher while the expression of TIMP-2 is significantly lower in CRC tissue in comparison to normal tissue [81]. Moreover, the authors found that the MMP-2/TIMP-2 ratio is higher in CRC tissue compared with healthy tissue, and decreases with tumor stage, depth of invasion and lymph node metastasis [81]. The expression of MMP-9 in CRC tissue is significantly higher in comparison to CA and normal mucosa, and correlates with tumor stage [82]. Moreover, enhanced MMP-9 expression in CRC cells is associated with increased invasiveness of the tumor [83], while levels of MMP-2 expression in CRC are lower than in adjacent normal mucosa, and significantly correlate with the depth of invasion and the presence of liver metastasis [84]. In addition, the overexpression of TIMP-1 correlates with the elevated expression of MMP-9, and may stimulate tumor growth and malignant transformation as well as inhibit tumor cell apoptosis [85,86,87].

Therefore, in our previous studies, which utilized the fast and easy-to-use ELISA method, we compared the clinical significance of both gelatinases (MMP-9 and MMP-2) as well as their tissue inhibitors (TIMP1 and TIMP-2, respectively) in the diagnosis of CRC as well as in the differentiation between CA and CRC. We determined concentrations of these proteins in the serum of patients with CRC in relation to the clinicopathological features of cancer and the serum levels of the classical tumor markers for CRC–CEA and CA 19-9. Moreover, we assessed the diagnostic criteria for all of the tested proteins. In our studies, the serum levels of MMP-9 and TIMP-1 were significantly higher while the concentrations of MMP-2 and TIMP-2 were statistically significantly lower in patients with CRC in comparison to healthy subjects [26,27,28]. Moreover, the serum TIMP-1 concentration was significantly elevated in patients with CRC in comparison to subjects with CA, and correlated with tumor stage, nodal involvement, the presence of distant metastases, patient survival, and tumor resectability [26]. These observations indicate that elevated TIMP-1 serum levels may reflect the role of this protein as a stimulator of tumor growth and malignant transformation, or an anti-apoptotic factor [86,87]. Our previous investigations have also demonstrated that serum MMP-2 and TIMP-2 concentrations decrease with tumor stage, while MMP-2 levels are significantly lower in patients with CRC in comparison to those with CA [27,28]. Our findings are consistent with the observations of other authors who have demonstrated elevated serum or plasma levels of MMP-9 in patients with CRC, and decreased plasma MMP-2 levels in CRC patients with metastatic liver disease in comparison to healthy controls [88,89]. An increased plasma level of TIMP-1 is a significant prognostic factor for the survival of patients with CRC [88,89,90,91]. In our other studies, we also evaluated the potential role of these proteins as biochemical markers for CRC. The diagnostic sensitivity of TIMP-1 was higher (61%) than that of the serum levels of other biomarkers (MMP-9—55%; TIMP-2—59%, MMP-2—46%) as well as the classical tumor markers (CEA and CA 19-9), and increased in combined use with CEA. Among all of the biomarkers tested, the highest AUC was found for the serum TIMP-1 levels [26,27,28]. Therefore, in our previous studies, we also compared the serum levels of MMP-2 and TIMP-2 in patients with CRC with their expression in cancer cells, inflammatory infiltrate cells and colorectal cells from adjacent normal tissue. We demonstrated that the percentages of positive immunoreactivity of these proteins were higher in malignant and inflammatory cells as compared to normal tissue. There were significant correlations between MMP-2 immunoreactivity in inflammatory cells and the presence of distant metastases, and between TIMP-2 expression in inflammatory cells and tumor size, nodal involvement and distant metastases [28]. The correlations were confirmed by the Spearman correlation test, which showed a significant positive association between the MMP-2 serum concentration and the expression of its inhibitor in the same types of cells, as well as between the serum levels of both proteins. Our findings suggest a complex role of the MMP-2/TIMP-2 network in CRC development and metastasis [28]. Furthermore, positive tissue expression of MMP-2 is a significant prognostic factor for the survival of patients with CRC, which has been confirmed by Langers et al. [92] who found that enhanced MMP-2 expression in normal colorectal mucosa is associated with reduced survival in patients with CRC.

Based on our results, we can suggest greater utility of serum TIMP-1 compared with MMP-9, MMP-2, and TIMP-2 in the diagnosis of CRC, particularly in the assessment of the tumor stage, survival of cancer patients, resectability of the tumor, and in the differentiation between CA and CRC. The presented findings indicate that selected MMPs and TIMPs play a role not only in tumor invasion and the initiation of metastasis, but also in carcinogenesis from colorectal adenomas [81]. Our results confirm a complex network of interactions between the tumor, its microenvironment, and stromal cells, but this issue requires further research [26,27,28].

## 5. Future Perspectives

The challenges facing medicine in the future lie in the establishment of new diagnostic strategies based on novel and accurate tumor biomarkers that will improve the early detection of malignant diseases, such as CRC, and facilitate differentiation between CRC and CA. A growing number of publications focus on the molecular and cellular mechanisms involved in the development, progression and metastasis of this malignancy. The design of epigenetic and genetic panels of biomarkers useful in CRC diagnosis constitutes a reasonable strategy in the clinical management of CRC [6,93,94]. There are three main mechanisms that are currently considered to be responsible for CRC pathogenesis. The first one is the suppressor pathway, or the pathway of chromosomal instability, which is associated with the accumulation of mutations leading to oncogene activation (*KRAS*) and suppressor gene inactivation (*TP53, DCC, SMAD4, APC*), and consequently to neoplastic transformation [5,95]. The second pathway is the accumulation of errors during DNA replication due to the presence of mutations in the genes responsible for its repair (*MLH1, MSH2, MSH6, PMS2, MLH3, MSH3, PMS1* and *Exo1*) [96]. The third mechanism is related to aberrant hypermethylation [97].

Many recent reports have focused on *RAS*, *BRAF* (Raf murine sarcoma viral oncogene homolog B) and *HER2* (human epidermal growth factor receptor 2 gene) mutations as predictive factors of mCRC patients who receive chemotherapy [98,99]. A study by Zheng investigated the frequency and prognostic role of *HER2* and *BRAF* gene mutations in CRC patients. The authors concluded that *HER2* amplification significantly correlates with greater bowel wall invasion and a more advanced TNM stage, while *HER2* amplification is an independent prognostic factor for worse disease-free survival [98]. Moreover, a statistically significant correlation for the RAS mutation and overall survival was also proved, whereas RAS mutation and liver metastasis were found to be independent factors for shorter overall survival of CRC patients in multivariate analysis [99].

A greater understanding of the pathways involved in CRC development will facilitate the establishment of diagnostic and prognostic biomarkers for this malignancy. In recent years, DNA and RNA markers in blood have been investigated as a potential diagnostic tool in CRC. It has been indicated that the analysis of biomarkers, such as DNA, RNA or proteins in the blood, accelerates the development of diagnostic tools in molecular biology. These techniques are characterized by greater sensitivity and enhanced cost-effectiveness, and may be employed in clinical practice [6].

## 6. DNA-Based Biomarkers

A variety of DNA markers have been assessed in plasma, including *APC, KRAS, p53, MLH1, HLTF, TMEF2, NGFR*, and *SEPT9* [6]. A study by Diehl et al., which utilized the detection of mutations by beads, emulsification, amplification and magnetics (BEAMing) assay, found that APC mutations in plasma samples were detected with a sensitivity of 73%, which, however, was limited to 9% in patients with CA [100]. Furthermore, some authors have demonstrated that the hypermethylation of *Septine 9* (guanosine triphosphatase class gene) is related to CRC development [101,102] and is found in 58–96% of CRC patients, and in only 18% of CA subjects with specificities of 86–100% [101,102,103,104].

## 7. RNA-Based Biomarkers

Some clinical investigations have revealed that the transcriptome of plasma and peripheral blood also offers potential diagnostic biomarkers [105,106]. A plasma biomarker panel including *BANK1, BCNP1, CDA, MGC20553* and *MS4A1* may discriminate patients with CRC from healthy subjects with a sensitivity and specificity of 88% and 64%, respectively [105,106]. Other molecular biomarkers provide a source of miRNAs [107,108,109]. Elevated miR92 levels have been detected in the plasma of patients with CRC compared with healthy individuals [110]. Moreover, statistically higher levels of miR92a and miR29a have been found in patients with CRC and CA in comparison to healthy controls [111].

## 8. Plasma Proteins

The development of new technologies in proteomics, such as chromatographic techniques based on mass spectrometry (MS) assays, surface-enhanced laser desorption/ionization time-of-flight (SELDI-TOF)-MS, and matrix-assisted laser desorption/ionization time-of-flight (MALDI-TOF) MS, allows for the identification of large-scale protein patterns. These methods allow for the identification of peptide patterns that discriminate patients with CRC from healthy individuals with a sensitivity and specificity of 90% [112]. However, they are not specific to CRC [113]. Some authors have evaluated the significance of epithelial cell adhesion molecules, p53, p62, CEA, HER-2/neu, Ras, topoisomerase Ⅱ-alpha, histone deacetylase 3 and 5, ubiquitin L3, tyrosinase, tropomyosin, and cyclin B1 as biomarkers for CRC. These stable biomarkers can be detected by immunoassays and are absent in healthy individuals, and therefore might be promising biomarkers for further research [6,114,115,116]. Moreover, some clinical investigations have assessed the combined ELISA analysis of MAPKAPK3 and ACVR2B in patients with CRC and healthy controls, with a sensitivity of 83% and a specificity of 74% [115,117].

Although there is still insufficient evidence supporting the use of biomarkers, such as genetic and epigenetic biomarker panels in CRC diagnosis, it appears to be a reasonable strategy for the medicine of the future [6]. However, what should also be taken into consideration is that CRC cells are able to enter blood via blood vessel invasion, where they circulate and release detectable biomarkers in the plasma or circulating phagocytes. Furthermore, such vessel invasion occurs more frequently in the advanced stages of CRC [118,119,120].

## 9. Conclusions

The World Health Organization predicts an increase of 77% in the number of newly diagnosed CRC cases, and an increase of 80% in the deaths from CRC by 2030 [6,121,122]. Thus, this disease is a global medical problem of profound significance. The establishment of new biochemical markers measured using low-cost, non-invasive techniques, which are easy to perform, is urgently required to improve the diagnosis of CRC and the treatment of patients with this malignancy. Biomarkers are a key tool in the early detection, survival and prognostication, and prediction of treatment responses. It has been indicated recently that a variety of specific proteins contribute significantly to tumor growth, dissemination, and local immune escape in the pathogenesis of many carcinomas, including CRC. However, little is known about the utility of selected circulating blood inflammatory mediators such as cytokines, including HCs and chemokines, and acute-phase protein—CRP—as well as various MMPs and TIMPs in patients with CRC. The present paper demonstrates that among all of the proteins tested in our investigations, serum M-CSF, CXCL-8, IL-6 and TIMP-1 are better biomarkers than the currently used, well-established classical tumor marker—CEA—in the diagnosis of CRC. Serum TIMP-1 measurement is useful in differentiating between CRC and CA. Moreover, serum M-CSF, CXCL-8, IL-6 and TIMP-1 levels correlate with CRC progression, and therefore are particularly useful in establishing tumor stage, distant metastases and/or nodal involvement, whereas the serum levels of M-CSF and CRP are independent prognostic factors for the survival of patients with CRC. This review confirms the promising significance of these proteins as circulating biomarkers for CRC. However, our findings need to be confirmed in future studies performed on a larger population of patients with CRC to ensure the reproducibility of the presented results, and to confirm their potential significance as novel biomarkers in CRC due to the non-specific nature of these proteins.

## Figures and Tables

**Table 1:** Clinical significance of selected biomarkers in the diagnosis and progression of CRC [18,19,20,21,22,23,24,25,26].

Clinical Significance	Biomarker	Findings	References
Diagnosis	SCF	Significantly lower serum levels between CRC and healthy individuals Diagnostic sensitivity higher than for classical tumor marker—CEA and CA 19-9	[19,21]
	M-CSF CXCL-8 IL-6 TIMP-1	M-CSF, CXCL-8, IL-6, TIMP-1—significantly higher serum levels between CRC and healthy individuals M-CSF—AUC higher than for classical tumor marker—CEA and CA 19-9 CXCL-8—AUC and diagnostic sensitivity higher than for classical tumor marker—CEA IL-6—AUC higher than for classical tumor marker—CEA and CA 19-9 TIMP-1—AUC and diagnostic sensitivity higher than for classical tumor marker—CEA and CA 19-9	[18,22,25]
Progression	M-CSF	Significant differences between serum levels and TNM stage serum levels and nodal involvement	[18,20]
	CXCL-8	Significant differences between serum levels and TNM stage serum levels and distant metastases	[22]
	IL-6	Significant differences between serum levels and TNM stage serum levels and nodal involvement serum levels and distant metastases	[25]
	CRP	Significant differences between serum levels and distant metastases	[25]
	TIMP-1	Significant differences between serum levels and TNM stage serum levels and nodal involvement serum levels and distant metastases	[26]
Independent prognostic factor	M-CSF	Higher serum levels as poor prognostic factor	[18]
	CRP	Higher serum levels as poor prognostic factor	[25]
CRC vs. CA differentiation	M-CSF	Significant differences between CRC and colorectal adenoma	[18]
	GM-CSF		[19]
	IL-6		[25]
	CRP		[25]
	TIMP-1		[26]

## Data Availability

Not applicable.
